# Development of a questionnaire on nutritional knowledge for the obese hospitalized patient: the NUTRIKOB questionnaire

**DOI:** 10.3389/fnut.2023.1232424

**Published:** 2023-07-18

**Authors:** Sara Paola Mambrini, Davide Soranna, Eva Averna, Giulia Di Guglielmo, Elisa Lucchetti, Tiziana Tinozzi, Calogero Vinci, Valerio Barbieri, Antonella Zambon, Simona Bertoli, Massimo Scacchi

**Affiliations:** ^1^International Center for the Assessment of Nutritional Status and the Development of Dietary Intervention Strategies (ICANS-DIS), Department of Food, Environmental and Nutritional Sciences (DeFENS), University of Milan, Milan, Italy; ^2^Laboratory of Metabolic Research, S. Giuseppe Hospital, Istituto Auxologico Italiano, IRCCS, Piancavallo, Italy; ^3^Biostatistic Unit, IRCCS Istituto Auxologico Italiano, Milan, Italy; ^4^Servizio di dietetica e nutrizione clinica -Ospedale San Giuseppe-Oggebbio- VB- Istituto Auxologico Italiano IRCCS, Oggebbio, Italy; ^5^Department of Statistics and Quantitative Methods, University of Milano-Bicocca, Milan, Italy; ^6^Lab of Nutrition and Obesity Research, Istituto Auxologico Italiano, IRCCS, Milan, Italy; ^7^Department of Clinical Sciences and Community Health, University of Milan, Milan, Italy

**Keywords:** obesity, nutritional knowledge, questionnaire, nutrition counseling, psychometric adequacy

## Abstract

**Introduction:**

Different approaches, involving different areas and figures, are useful for the rehabilitation of obese subjects through a multidisciplinary hospital path. A focal point of rehabilitation is represented by education on healthy eating by increasing the dietary knowledge patients. Few tools investigating food knowledge are available in Italy: therefore, the need has emerged to develop easy-to-use tools for clinical practice that allow to detect food knowledge to set up a more targeted food re-education. The following work aimed at building and validating a questionnaire capable of investigating the dietary knowledge of the population affected by obesity.

**Methods:**

A pool of experts carried out a review of the literature, gathering all the information necessary to select and construct the best set of questions and the format of the final project of the questionnaire. During statistical analysis the validity, reproducibility and stability of the questionnaire were investigate in a sample of 450 subjects with obesity.

**Results:**

Early analysis disclosed that 5 questions of the original questionnaire had no discriminating power. The successive validation phases were successful, confirming good content validity, stability and reproducibility over time.

**Discussion:**

The questionnaire has all the characteristics to be considered a valid tool for investigating dietary knowledge in the obese population. The psychometric tests confirmed a good internal consistency of the structure, a validity of the content, a good reproducibility and stability over time.

## 1. Introduction

Nutritional knowledge is the set of concepts concerning nutrition, based on national and international guidelines. It has been shown that nutritional knowledge is a relevant part of the decision-making process in choosing foods, along with other factors such as age, gender and socioeconomic status (1, 2). In fact, nutritional knowledge can influence food choices both indirectly, e.g., by helping to understand and memorize the reading of food labels, and directly on consumer behavior (1, 3, 4). Recent studies have shown that limited health literacy, of which nutritional knowledge is a part, is significantly correlated with increased onset of type 2 diabetes and cardiovascular diseases (5). It is also known that many non-communicable diseases are linked to the diet: excessive consumption of highly processed foods, with high sugar or saturated fats content, is correlated with an increased risk of obesity, cardiovascular diseases, and cancer (6).

This makes it clear how important it is to pay more attention to nutritional education and to monitoring nutritional knowledge over time, especially for certain target populations, such as the population suffering from overweight or obesity. (7, 8). This is even more relevant when nutritional education becomes part of the therapeutic approach, such as for patients with severe obesity (8, 9). In obese and overweight populations, a higher level of nutritional knowledge, achieved through nutritional education, has been shown to be associated with greater weight loss. This is explained by increased awareness in food choices leading to better nutritional quality and improvement in food habits and patient empowerment (10–13). Measurement of nutrition knowledge is challenging. Most studies have used written questionnaires, but many of these have inadequate or no validation (14–16). In 1999, Parmenter and Wadler developed and validated, according to psychometric standards, the General Nutritional Knowledge Questionnaire (GNKQ), which investigates nutritional knowledge in the UK population, exploring the following four points: 1) expert recommendations 2) knowledge about nutrient-source foods 3) correct food choices 4) the relationship between diet and disease (17). In 2016, an update of the GNKQ to the latest guidelines was carried out (18). Various validations of the GNKQ for different countries are available, based on that country’s guidelines and also on cultural adaptation of food (19–21). In 2007 Moynihan validated, based on the GNKQ, a shorter questionnaire that was able to investigate the nutritional knowledge of the elderly population living in sheltered housing (22). This instrument has the advantage of being easier to use in clinical practice but suffer from limitations in the topic areas investigated. In any case, because of its clinical practicality in 2010 Vico et al. validated the Italian version of the Moynihan questionnaire (23, 24). To date, this version remains the one of the few validated questionnaires for the investigation of nutritional knowledge in Italian language. These instruments have the capability of investigating nutritional knowledge in the general population. Few questionnaires, however, have been validated to specifically investigate nutritional knowledge in the obesity population. The only instrument in the literature is the questionnaire constructed by Feren et al. in 2011 (25). Taking over the structure and content of the GNKQ, the authors made a cultural adaptation to the traditions of the Nordic countries. Based on the above considerations, we believed it was important to construct a questionnaire based on the most up-to-date Ministry of Health guidelines and centered on the principles of the Mediterranean diet, which is also able to cope with false food myths (26). In doing so we also took into account the psychometric reference guidelines (27). In larger scale, this instrument might also help to focus nutrition education aimed at correcting misconceptions and giving the necessary tools for patient empowerment. The aim of this work was to construct and validate a questionnaire investigating nutritional knowledge in adult individuals with obesity and wish the potential of being a useful tool during clinical practice to monitor the results of nutritional rehabilitation in this population.

## 2. Patients and methods

### 2.1. Participants

We recruited patients hospitalized in San Giuseppe Hospital of Piancavallo, Istituto Auxologico Italiano (Verbania, Piedmont, Italy) during the period April–October 2019. We included the patients with BMI ≥ 30 Kg/m^2^ and with age between 18 and 75 years. To make data relevant to the study aim, we excluded (i) foreign patients who did not understand the Italian language, (ii) illiterate patients and (iii) patients with learning disabilities. For the pilot phase we included a convenience sample of 100 obese patients hospitalized at Piancavallo. For the conclusive phase we included other 350 patients for a total of 450 patients. Subjects were enrolled in the first few days of admission to the facility before the start of the nutritional rehabilitation program.

Moreover, we analyzed 300 students (150 enrolled in the third year of the Nutrition degree and 150 enrolled in the third year of the Psychology degree of the University of Milan) for construct validity analysis. The study protocol was approved by Ethics Committee in 19/01/2019 (18C901_2019) and informed consent was obtained from all participants.

The first step of the questionnaire development process consisted in studying the theory from which the phenomenon originates and in identifying aspects to which it might be related. Investigation of the theory was carried out through consultation of specific questionnaire construction guidelines and specific guidelines addressing the role of nutritional knowledge in the management of obesity (28).

A review of the scientific literature of the last 10 years was carried out using the keywords “questionnaire,” “nutritional knowledge,” “obesity” to search for already published questionnaires concerning education in the field of nutrition. Later, we created the first draft of questionnaire by means of (i) the identification of several items from published questionnaires related to nutritional education, (ii) the creation of items based on the experience of the researchers involved in the study. Subsequently this first draft was submitted to a panel of experts (clinical endocrinologists and nutritionists and health professionals) to optimize the sequence of items.

The analysis of item characteristics and measurement properties of the questionnaire was conducted in two phases: pilot and conclusive.

### 2.2. Method

#### 2.2.1. Item difficulty analysis

An item was not considered useful if it was answered correctly by more than 90% or less than 15% of individuals (29). Items that met these criteria were excluded, except if they were considered essential for content validity by the experts.

#### 2.2.2. Internal consistency analysis

The Kuder Richardson-20 (KR-20), the version of Cronbach’s α for dichotomous item, was calculated. KR-20 is a measure of internal consistency, that is, how closely related a set of items are as a group. A “high” value for KR-20 does not imply that the measure is unidimensional.

#### 2.2.3. Construct validity

We evaluated the construct validity (if the questionnaire reflects the true theoretical meaning of the construct of interest) comparing the score obtained in two groups: one which is known to have “good nutrition knowledge” and the other which has not. The score of the “good nutrition knowledge” group should be significantly higher when evaluated by an independent sample’s t-test or Wilcoxon test (25).

#### 2.2.4. Test and retest

A subsample was asked to recompile the questionnaire 2 weeks later. A test–retest approach was applied to the measured data to assess the stability of the instrument: specifically, the Pearson (or Spearman) correlation index was calculated, and it was considered a good measure of stability if the index score was greater than 0.7.

#### 2.2.5. Dimensional validation and reduction of items

Rasch analysis is a known psychometric technique used to assess whether a single latent trait drives item responses in a questionnaire (30). This model assumes that the probability of a correct response is determined by the item’s difficulty/discrimination and by the subject’s ability. The unidimensional assumption suggests that the correlation among items can be explained by a single latent factor. To verify this hypothesis, we evaluated the eigenvalues of polychromic correlation matrix among items. If the first eigenvalue is much greater than the others a unidimensional model is reasonable. Moreover, the model estimates the difficulty and the discriminant performance of each item. Negative values of regression coefficient related to difficulty suggest items relatively easy, useful in discriminating subjects who have lower abilities. Small values (<0.5) of regression coefficient related to discrimination suggest that the corresponding item is not a good indicator.

#### 2.2.6. Discrimination performance

**Figure 1:**
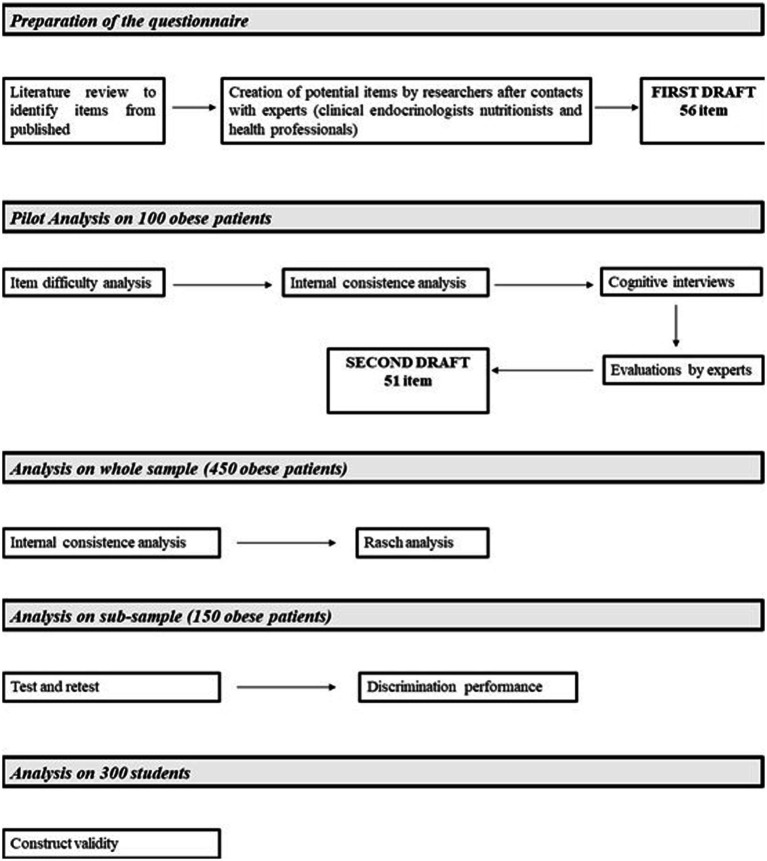
Flow chart describing the different stages of questionnaire construction and validation.

A subsample of patients was invited to participate in an interview with one or more clinical experts who classified each patient, based on his/her dietary knowledge, into two categories: “good knowledge of nutrition “and “no good knowledge of nutrition.” The clinical expert’s judgment was considered the gold standard. A ROC analysis was conducted to assess the discriminating power of the questionnaire (AUC >0.7 was considered as good discrimination) and the cut-off that best discriminated “good” knowers from “not good” was identified using the Youden index method. Figure 1 shows the flow chart of the construction and validation steps.

## 3. Results

The review of the literature revealed that the General Nutritional Knowledge Questionnaire (GNKQ), by Parmenter and Wardle, has often been used as a model for the development of different questionnaires to investigate nutritional knowledge; our focus was therefore on European adaptations of the GNKQ. The questionnaire makes it possible to probe areas of concern regarding healthy eating, highlighting the relationship between dietary knowledge and eating behavior. Drawing on the results obtained from the literature review and the Moynihan questionnaire, already in use at the Istituto Auxologico Italiano, a questionnaire was elaborated that took into account the basic structure of the GNKQ, the Norwegian Questionnaire, and the Moynihan questionnaire, and that took into consideration the typology of severe obese patients. In this way, items of interest potentially useful for the measurement of the survey construct in the study were identified and others were included based on the experience of the researchers involved. During this phase, particular attention was paid to establishing a logical sequence of the topics covered, in particular by identifying four sessions, and to the sequence of the individual questions comprising each section. Based on our experience with the structuring of the questions, we decided to use only closed, single-choice questions that could be easily understood by the patient. Particular care was also taken with regard to cultural and linguistic adaptation, compared to the questions taken as examples from the GNKQ and Norwegian questionnaires, by replacing foods typical of Nordic cuisine with those of the Mediterranean tradition. For the drafting of the questionnaire, the experts also relied on the most recent guidelines for a healthy diet drawn up by the Italian Ministry of Health. The first draft of the questionnaire was designed as a structured scale with 56 items. Each item was assigned a value of 1 if the answer was correct and 0 if the answer was wrong or missing. The score of the questionnaire was given by the sum of the values assigned to each response. The average compiling time was estimated to be 20 min.

### 3.1. Pilot analysis

#### 3.1.1. Item difficulty analysis

The draft questionnaire was submitted to the first 100 obese patients included in the sample. The mean age was 52 years, the 39% was male and mean BMI was 45 Kg/m^2^. The item difficulty analysis highlighted that 5 items had a correctness rate higher than 90% or below 15%. So the new version of the questionnaire was composed by 51 items.

#### 3.1.2. Internal consistency analysis

The KR-20 resulted 0.83 (95% CI 0.77–0.87) suggesting high internal consistency. When each item was removed, we observed a relative KR-20 value very similar to the value calculated on all items.

#### 3.1.3. Dimensional validation and reduction of items

**Figure 2:**
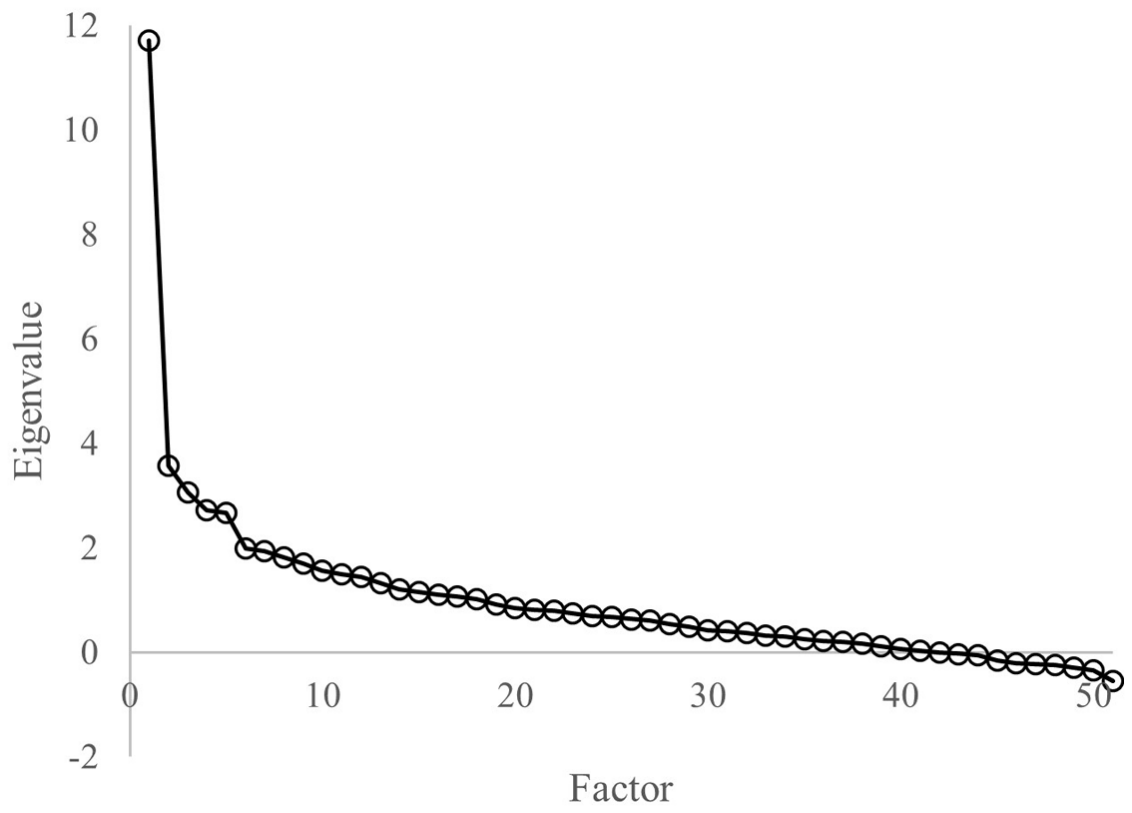
Eigenvalues of the polychoric correlation matrix in pilot study.

Figure 2 shows that the first eigenvalue was much greater than the others suggesting that a unidimensional model was reasonable. Although several items showed low discrimination ability we postponed this evaluation because results from small samples could lead to opposite conclusions compared with those based on larger samples (31).

### 3.2. Analysis on whole sample

#### 3.2.1. Internal consistency analysis

For this analysis we considered the whole sample of 450 obese patients (mean age of 51 years, 43% of male and mean BMI equal to 46 Kg/m^2^). The KR-20 value was confirmed (0.81, 95% CI 0.78 to 0.83). As in the pilot phase, when each item was removed, we observed that the relative KR-20 value was very similar or lower than KR-20 calculated on all items.

#### 3.2.2. Dimensional validation and reduction of items

**Figure 3:**
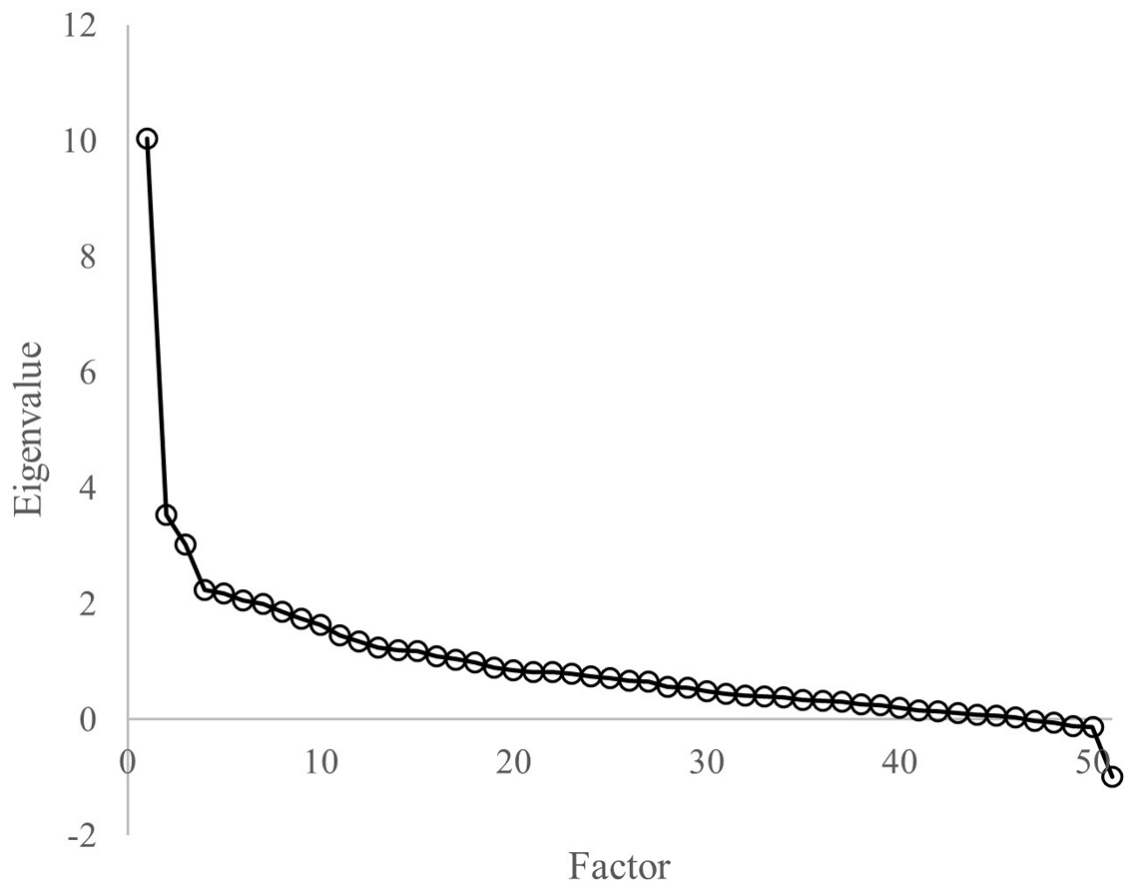
Eigenvalues of the polychoric correlation matrix in whole simple.

Figure 3 shows that the first eigenvalue was much greater than the others suggesting that a unidimensional model was reasonable. The analysis identifies several potential items (6, 7, 11, 12, 19, 20, 23, 29, 32, 36, 37, 44) with low discrimination ability varying from −0.20 to 0.46 (see Table 1).

**Table 1:** Rasch analysis results.

Item	Difficulty coefficient	Discrimination coefficient
6	−0.45193	0.1586
7	−2.64031	0.40154
11	0.39381	0.37241
12	−4.55899	0.17564
19	−5.42843	−0.20317
20	−5.14404	0.16161
23	−1.04882	0.39225
29	−0.95737	0.2364
32	−2.25652	0.46481
36	1.90114	0.25648
37	3.63235	0.1433
44	−3.78028	0.15048

#### 3.2.3. Construct validity

We submitted the final draft to 150 nutrition students and 150 psychology students. The nutrition students’ score was significantly higher (value of *p* <0.0001) than the one obtained by psychology students (median 50.00 [35.00 to 51.00] vs. 35.00 [30.00 to 38.00] respectively).

#### 3.2.4. Test and retest

In a sub-sample of 150 obese patients (mean age 50 years, 45% males and mean BMI 46 Kg/m^2^) the questionnaire was re-submitted after 2 weeks. The Spearman correlation of the two scores was 0.80 (95% CI 0.73 to 0.85).

#### 3.2.5. Discrimination performance

Finally, for 143 patients of the sub-sample used for test–retest analysis we had the information of the expert clinicians classification: 99 classified as “good knowledge of nutrition” (69%) and 44 as “no good knowledge of nutrition” (31%). The median score in “good knowledge of nutrition” was 37.10 and in “no good knowledge of nutrition” was 29.68. The AUC value of score was 0.798, suggesting a good discrimination performance. Moreover, the threshold of 35 (identified by Youden index) was characterized by sensitivity of 70% and specificity of 77%.

## 4. Discussion

The aim of this work was to develop and validate a new questionnaire to investigate nutritional knowledge in a population with severe obesity. This is necessary because nutritional education and hence knowledge are an integral part of the process of treatment and management of this disease. As already stated in the literature, a few validated questionnaires are available that can correctly investigate the construct of nutritional knowledge and that are up to date with the latest guidelines. Furthermore, it is important to emphasize that an instrument is often defined on a specific population and is therefore not very adaptable to other populations.

One questionnaire is used in Italy, validated in Italian, for adult people by the original version of the Moynihan questionnaire. This questionnaire, however, is poorly suited to specifically investigate the population affected by obesity. Although this questionnaire is useful in clinical practice, it lacks some important topics, such as simple sugars or reading food labels. Furthermore, the combination of closed or fill-in and open questions is not always easy to understand. The use of an inverse score (e.g., a high score corresponds to low nutritional knowledge) is also not always easy to understand.

### 4.1. Pilot study

The initial pool of items was made up of items already present in validated instruments, to which items were added to incorporate content deemed important for the target population by the panel of experts. This resulted in an initial draft of 56 questions. For a better investigation of food knowledge, it was decided to divide the questions into four sections: (i) expert recommendations; (ii) food groups and nutrients; (iii) healthy food choices; (iv) the relationship between diet and health and disease risk. The analysis in the pilot study of internal consistency also yielded positive results, stating a good internal consistency of both the individual sections of the questionnaire and the entire item pool. This result has also been confirmed in the analysis of the full sample (450 patients).

### 4.2. Whole sample

From Rasch analysis, emerged that some items did not have a high discriminatory capacity of the construct. The experts carefully evaluated the questions and considered them to be of importance within the overall framework of nutritional knowledge and for the nutritional rehabilitation of the patient. In particular, the experts decided to keep the questions concerning the consumption of fruit and vegetables and the fat content of some foods or limiting the consumption of sugar. These questions are considered essential to assess nutritional knowledge, closely related to the dietary recommendations drawn up by the ministry. The questionnaire, after the elimination of non-discriminating items, consists of 51 items (see Appendix 1, 2).

By administering the questionnaire to nutrition students and psychology students, we were able to assess content validity. The nutrition students scored higher than the psychology students, supporting the fact that the questionnaire can correctly induct the construct, i.e., different dimensions of nutrition knowledge. These data are in agreement with findings from previous questionnaire validation studies (17, 25). Two weeks after the first administration, the questionnaire was re-administered to the subjects to assess the stability of the items over time. The Spearman correlation coefficient returned a good stability of the questionnaire for all items, confirming a consistency of measurement over time. Similar results were found by other researchers during the validation of other questionnaires (17, 19, 25, 32). Finally, the discrimination performance test returned an AUC of 0.78, confirming a good ability of the instrument to investigate the construct. It was also possible to identify a scoring threshold of 35 as a discriminator between subjects classified as good knowers (score > 35) and non-knowers (score < 35). To date, given the limited number of subjects, it is not possible for us to obtain a cut-off scale discriminating various degrees of knowledge in adequately robust way. We provide for the creation of a scoring scale that allows us to better classify the. The widening of the sample of patient will likely allow us to better classifying results obtained through the completion of the questionnaire into different sub-categories.

A strength of this work is the sample size of both the pilot study (100) and the other phases (450), which is higher than in other validation studies (17, 21, 23). The reliability of the test depends on the sample size and test length (33).

### 4.3. Limits

A limitation of this questionnaire can be found in the number of questions, which can lead to excessive length. It was observed that the average filling-in time was approximately 30 min. We believe that, despite this completion time, the questionnaire is usable in clinical hospital practice. We also consider the possibility of creating a simplified version that would consider a smaller number of questions.

## 5. Conclusion

Based on the results obtained, we propose to the scientific and clinical communities engaged in management of obesity the use of this new tool, representing a helpful indicator

of the patient’s treatment pathway and of the impact exerted by nutritional rehabilitation. In agreement with the most up-to-date guidelines, placing nutritional education of the obese patient at the center, the NUTRIKOB questionnaire is effective as a means of monitoring nutritional education in this clinical condition.

## Data availability statement

The raw data supporting the conclusions of this article will be made available by the authors, without undue reservation.

## Ethics statement

The studies involving human participants were reviewed and approved by Istituto Auxologico Italiano Ethics Committee. The patients/participants provided their written informed consent to participate in this study.

## Author contributions

SM and DS: conceptualization, data curation, and project administration. DS, AZ, and SM: methodology and writing original draft. DS and AZ: formal analysis. EL, EA, GG, TT, CV, VB, and SM: resources. MS, SB, and AZ: writing review and editing. MS, SB, and VB: supervision. All authors contributed to the article and approved the submitted version.

## Funding

This work was supported by Italian Ministry of Health–Ricerca Corrente.

## Conflict of interest

The authors declare that the research was conducted in the absence of any commercial or financial relationships that could be construed as a potential conflict of interest.

## Publisher’s note

All claims expressed in this article are solely those of the authors and do not necessarily represent those of their affiliated organizations, or those of the publisher, the editors and the reviewers. Any product that may be evaluated in this article, or claim that may be made by its manufacturer, is not guaranteed or endorsed by the publisher.

## References

[1] Worsley A. Nutrition knowledge and food consumption: can nutrition knowledge change food behaviour? Asia Pac J Clin Nutr. (2002) 11:S579–85. doi: 10.1046/j.1440-6047.11.supp3.7.x 12492651

[2] Koch F Hoffmann I Claupein E. Types of nutrition knowledge, their socio-demographic determinants and their association with food consumption: results of the NEMONIT study. Front Nutr. (2021) 8:1–11. doi: 10.3389/fnut.2021.630014, PMID: 33644108 PMC7907003

[3] Miller LMS Cassady DL. The effects of nutrition knowledge on food label use. A review of the literature. Appetite. (2015) 92:207–6. doi: 10.1016/j.appet.2015.05.029, PMID: 26025086 PMC4499482

[4] Leng G Adan RAH Belot M Brunstrom JM De Graaf K Dickson SL. The determinants of food choice. Proc Nutr Soc. (2017) 76:316–7. doi: 10.1017/S002966511600286X 27903310

[5] Williams MV Baker DW Parker MD. Relationship of functional health literacy to patients’ knowledge of their chronic disease. Origin Invest. (1998) 158:166–2. doi: 10.1001/archinte.158.2.166 9448555

[6] Nishida C Uauy R Kumanyika S Shetty P. The joint WHO/FAO expert consultation on diet, nutrition and the prevention of chronic diseases: process, product and policy implications (2003) 2004:245–07. 10.1079/phn2003592 14972063

[7] Wang L Zhuang J Zhang H Lu W. Association between dietary knowledge and overweight/obesity in Chinese children and adolescents aged 8-18 years: a cross-sectional study. BMC Pediatr. (2022) 22:558. doi: 10.1186/s12887-022-03618-2, PMID: 36138367 PMC9502888

[8] Valmórbida JL Goulart MR Busnello FM Pellanda LC. Nutritional knowledge and body mass index: a cross-sectional study. Rev Assoc Med Bras. (2017) 63:736–07. doi: 10.1590/1806-9282.63.09.736 29239470

[9] Ryan DH Kahan S. Guideline recommendations for obesity management. Med Clin North Am. (2018) 102:49–63. doi: 10.1016/j.mcna.2017.08.006 29156187

[10] Klohe-Lehman DM Freeland-Graves J Anderson ER McDowell T Clarke KK Hanss-Nuss H. Nutrition knowledge is associated with greater weight loss in obese and overweight low-income mothers. J Am Diet Assoc. (2006) 106:65–75. doi: 10.1016/j.jada.2005.09.047, PMID: 16390668

[11] Spronk I Kullen C Burdon C Connor HO. Systematic review relationship between nutrition knowledge and dietary intake (2014) 111:1713–26. doi: 10.1017/S0007114514000087 24621991

[12] Balani R Herrington H Bryant E Lucas C Kim SC. Nutrition knowledge, attitudes, and self-regulation as predictors of overweight and obesity (2019) 31 10.1097/JXX.0000000000000169 30829975

[13] Gilardini L Cancello R Caffetto K Cottafava R Gironi I Invitti C. Nutrition knowledge is associated with greater weight loss in obese patients following a multidisciplinary rehabilitation program. Minerva Endocrinol (Torino). (2021) 46:296–302. doi: 10.23736/S2724-6507.20.03212-5 32720499

[14] Parmenter K Waller J Wardle J. Demographic variation in nutrition knowledge in England. Health Educ Res. (2000) 15:163–4. doi: 10.1093/her/15.2.163, PMID: 10751375 PMC4344545

[15] Siegrist M. Consumers’ knowledge of healthy diets and its correlation with dietary behaviour (2011) 24:54–60. doi: 10.1111/j.1365-277X.2010.01124.x 20880377

[16] Trakman GL Forsyth A Devlin BL Belski R. A systematic review of athletes’ and coaches’ nutrition knowledge and reflections on the quality of current nutrition knowledge measures. Nutrients. (2016) 8. doi: 10.3390/nu8090570, PMID: 27649242 PMC5037555

[17] Parmenter K Wardle J. Development of a general nutrition knowledge questionnaire for adults. Eur J Clin Nutr. (1999) 53:298–8. doi: 10.1038/sj.ejcn.1600726 10334656

[18] Kliemann N Wardle J Johnson F Croker H. Reliability and validity of a revised version of the general nutrition knowledge questionnaire. Eur J Clin Nutr. (2016) 70:1174–80. doi: 10.1038/ejcn.2016.87, PMID: 27245211 PMC5014128

[19] Gao Z Wu F Lv G Zhuang X Ma G. Development and validity of a general nutrition knowledge questionnaire (Gnkq) for Chinese adults. Nutrients. (2021) 13. doi: 10.3390/nu13124353, PMID: 34959905 PMC8707636

[20] Thompson C Vidgen HA Gallegos D Hannan-jones M. Validation of a revised general nutrition knowledge questionnaire for Australia (2020) 24:1608–18. doi: 10.1017/S1368980019005135 PMC10200366 32383425

[21] Bukenya R Ahmed A Andrade JM Grigsby-Toussaint DS Muyonga J Andrade JE. Validity and reliability of general nutrition knowledge questionnaire for adults in Uganda. Nutrients. (2017) 9:1–11. doi: 10.3390/nu9020172 PMC5331603 28230779

[22] Moynihan PJ Mulvaney CE Adamson AJ Seal C Steen N Mathers JC. The nutrition knowledge of older adults living in sheltered housing accommodation. J Hum Nutr Diet. (2007) 20:446–8. doi: 10.1111/j.1365-277X.2007.00808.x, PMID: 17845379

[23] Da Vico L Biffi B Agostini S Brazzo S Masini ML Fattirolli F. Validation of the Italian version of the questionnaire on nutrition knowledge by Moynihan. Monaldi Arch Chest Dis Card Ser. (2010) 74:140–6. 10.4081/monaldi.2010.263 21114058

[24] Rosi C Pennella S Fantuzzi AL Pedrazzi P Passalacqua M Gavioli M. The usefulness of Moynihan questionnaire in the evaluation of knowledge on healthy diet of patients undergoing cardiology rehabilitation. Monaldi Arch Chest Dis Card Ser. (2013) 80:76–89. 10.4081/monaldi.2013.83 24494411

[25] Feren A Torheim LE Lillegaard ITL. Development of a nutrition knowledge questionnaire for obese adults. Food Nutr Res. (2011) 55:7271–7. doi: 10.3402/fnr.v55i0.7271 PMC3193827 22007155

[26] Florença SG Ferreira M Lacerda I Maia A. Food myths or food facts? Study about perceptions and knowledge in a Portuguese sample. Foods. (2021) 10. doi: 10.3390/foods10112746 PMC8623929 34829026

[27] Trakman GL Forsyth A Hoye R Belski R. Review article developing and validating a nutrition knowledge questionnaire: key methods and considerations (2017) 20:2670–9. doi: 10.1017/S1368980017001471 PMC10261290 28735598

[28] Boateng GO Neilands TB Frongillo EA Melgar-Quiñonez HR Young SL. Best practices for developing and validating scales for health, social, and behavioral research: a primer. Front Public Heal. (2018) 6:1–18. doi: 10.3389/fpubh.2018.00149 PMC6004510 29942800

[29] Domino G Domino M. Psychological Testing. 2nd edn. Cambridge University Press. (2006). Available at: https://www.perlego.com/book/1693615/psychological-testing-an-introduction-pdf (Accessed: 14 October 2022).

[30] Batchelder L Fox D Potter CM Peters M Jones K Forder JE. Rasch analysis of the long - term conditions questionnaire (LTCQ) and development of a short - form (LTCQ - 8). Health Qual Life Outcom. (2020) 18:1–12. doi: 10.1186/s12955-020-01626-3, PMID: 33256754 PMC7706038

[31] Schutte L Wissing MP Ellis SM Jose PE Vella-brodrick DA. Rasch analysis of the meaning in life questionnaire among adults from South Africa, Australia, and New Zealand. Health Qual Life Outcom. (2016) 14:1–15. doi: 10.1186/s12955-016-0414-x, PMID: 26790952 PMC4719730

[32] Matsumoto M Tanaka R Ikemoto S. Validity and reliability of a general nutrition knowledge questionnaire for Japanese adults. J Nutr Sci Vitaminol (Tokyo). (2017) 63:298–5. doi: 10.3177/jnsv.63.298, PMID: 29225314

[33] Paul K. The handbook of psychological testing. 2nd ed. Milton Park: Taylor & Frances/Routledge (1993).

