# Supplementary material for: Development of a questionnaire on nutritional knowledge for the obese hospitalized patient: the NUTRIKOB questionnaire
Source: Front Nutr. 2023 Jul 18;10:1232424. doi: 10.3389/fnut.2023.1232424 (PMC10400352; doi:10.3389/fnut.2023.1232424)
Supplement: Supplementary file 1 [file Data_Sheet_1.PDF]

Questionario di conoscenza generale sulla nutrizione

-NUTRIKOB-

Nome……………………………………….Cognome…………………………………..Data nascita……………..

Data di compilazione…………………

1 Sezione 1: le raccomandazioni degli esperti

1. Qual è la frequenza raccomandata per il consumo dei seguenti alimenti? (una risposta per ogni alimento)

	quotidiano	settimanale	occasionale	non lo so
Bevande con zuccheri aggiunti [1]	□	□	□	□
Verdura [2]	□	□	□	□
Salse (es. maionese) [3]	□	□	□	□
Carne rossa [4]	□	□	□	□
Alimenti integrali [5]	□	□	□	□
Formaggi e insaccati [6]	□	□	□	□

2. Quale latte o yogurt è preferibile consumare? (una sola risposta) [7]

Intero	□
A ridotto contenuto di grassi	□
Entrambe le risposte precedenti	□
Nessuno dei due, i latticini dovrebbero essere evitati	□
Non lo so	□

3. Quante volte alla settimana è raccomandato il consumo di pesce? (una sola risposta) [8]

1-2 volte a settimana □
3-4 volte a settimana □
Ogni giorno □
Non lo so □

4. Quante volte alla settimana è raccomandato il consumo della prima colazione? (una sola risposta) [9]

3 volte alla settimana □
4 volte alla settimana □
Ogni giorno □
Non lo so □

5. Quanti pasti è consigliato consumare ogni giorno? (una sola risposta) [10]

1-2 □
3-5 □
6-7 □
Non lo so □

6. In una dieta equilibrata quali pasti devono prevedere il consumo di proteine vegetali o animali? (una sola risposta) [11]

Colazione, pranzo e cena □
Solo cena □
Colazione e pranzo □
Non lo so □

2 Sezione 2: gruppi alimentari e nutrienti

1. Come valuti il contenuto di zuccheri aggiunti di questi alimenti? (una risposta per ogni alimento)

	Alto	Basso	Non lo so
Bevande zero o light [12]	□	□	□
Confettura di frutta [13]	□	□	□
Yogurt al naturale [14]	□	□	□
Ketchup [15]	□	□	□
Melone [16]	□	□	□

2. Come valuti il contenuto di sale di questi alimenti? (una risposta per ogni alimento)

	Alto	Basso	Non lo so
Frutta [17]	□	□	□
Pane in cassetta [18]	□	□	□
Ricotta [19]	□	□	□
Carne [20]	□	□	□
Alimenti in scatola (tonno-legumi) [21]	□	□	□

3. Come valuti il contenuto di fibra di questi alimenti? (una risposta per ogni alimento)

	Alto	Basso	Non lo so
Kiwi [22]	□	□	□
Noci [23]	□	□	□
Carne bianca [24]	□	□	□
Uova [25]	□	□	□
Lenticchie [26]	□	□	□
Pasta comune [27]	□	□	□

4. Pensi che questi alimenti siano una buona fonte di proteine? (una risposta per ogni alimento)

	Si	No	Non lo so
Pollo [28]	□	□	□
Formaggio [29]	□	□	□
Frutta [30]	□	□	□
Fagioli [31]	□	□	□
Burro [32]	□	□	□
Pesce [33]	□	□	□

5. Le vitamine e i sali minerali apportano la stessa quantità di calorie rispetto a carboidrati e proteine: (una sola risposta) [34]

Vero □
Falso □
Non lo so □

6. La quantità di calcio in un bicchiere di latte intero paragonato ad un bicchiere di latte scremato è (una sola risposta) [35]:

Circa lo stesso □
Più alto □
Più basso □
Non lo so □

7. Quali dei seguenti nutrienti ha maggior contenuto calorico a parità di peso? (una sola risposta) [36]

Zuccheri □
Carboidrati □
Proteine □
Grassi □
Non lo so □

8. Ad una persona che necessita di perdere peso consiglieresti l'utilizzo di integratori di sali minerali e vitamine [37]:

Vero □
Falso □
Non lo so □

3 Sezione 3: le scelte salutari degli alimenti

1. Quale tra le seguenti potrebbe essere una scelta migliore per limitare i grassi consumando un pasto al ristorante? (una sola risposta) [38]

   Tortellini panna e prosciutto □
   Risotto ai quattro formaggi □
   Gnocchi al ragù □
   Non lo so □

2. Quale tra le seguenti potrebbe essere una scelta migliore per un pasto completo? (una sola risposta) [39]

   Arrosto di tacchino, purea di patate e verdure □
   Spezzatino con piselli e patate arrosto □
   Frittura di pesce □
   Non lo so □

3. Quale tra le seguenti potrebbe essere una scelta migliore per un "pasto veloce"? (una sola risposta) [40]

   Trancio di pizza e bevanda alla cola + frutta □
   Panino con prosciutto cotto e formaggio + yogurt magro + acqua □
   Panino con insalata e tonno + spremuta + acqua □
   Non lo so □

4. Nella lista degli ingredienti il primo dell’elenco è sempre quello contenuto in maggiore quantità: (una sola risposta) [41]

Vero □
Falso □
Non lo so □

5. I cibi light sono sempre una scelta migliore [42]

Vero □
Falso □
Non lo so □

6. Quale tra i seguenti prodotti caseari potrebbe essere una scelta migliore se si vuole ridurre la quantità di grassi? (una sola risposta) [43]

Mozzarella light □
Formaggio spalmabile □
Fiocchi d latte □
Non lo so □

7. Tra queste due creme spalmabili alla nocciola quale contiene un maggior quantitativo di zucchero? (una sola risposta) [44]

**CREMA DI NOCCIOLA A**
zucchero, olio di palma, nocciole cacao magro,latte scremato in polvere, lattosio, siero del latte in polvere, emulsionante: lecitina (soia), vanillina

**CREMA DI NOCCIOLA B**
nocciole, zucchero di canna, cacao amaro.

Crema A □
Crema B □
Non lo so □

4 Sezione 4: relazione tra alimentazione, salute e rischio di malattie

1. Quale di queste condizioni è correlata ad un basso consumo di fibra alimentare? (una sola risposta) [45]

   Disordini intestinali ◻
   Anemia ◻
   Carie dentale ◻
   Non lo so ◻

2. Quale di queste patologie è correlata ad un elevato consumo di sale? (una sola risposta)

   [46]Ipotiroidismo ◻
   Diabete di tipo 1 ◻
   Ipertensione ◻
   Non lo so ◻

3. Quale di queste raccomandazioni aiuta a prevenire le malattie cardiovascolari? (una sola risposta) [47]

   Assumere integratori multivitaminici ◻
   Mangiare meno pesce azzurro ◻
   Mangiare meno grassi saturi ◻
   Non lo so ◻

4. Mangiare alimenti senza glutine aiuta nel controllo del peso [48]:

   Vero ◻
   Falso ◻
   Non lo so ◻

5. Quale di queste raccomandazioni aiuta a prevenire il diabete alimentare (diabete di tipo 2)? (una sola risposta) [49]

   Mangiare meno cibi raffinati (farina bianca, zucchero bianco) ◻
   Bere più succo di frutta ◻
   Mangiare più insaccati ◻
   Non lo so ◻

6. Mangiare formaggio senza lattosio aiuta a controllare i livelli di colesterolo nel sangue [50]:

Vero	□
Falso	□
Non lo so	□

7. Ad una persona che necessita di perdere peso consiglieresti una dieta senza glutine [51]:

Vero	□
Falso	□
Non lo so	
