# Supplementary material for: Development of a questionnaire on nutritional knowledge for the obese hospitalized patient: the NUTRIKOB questionnaire
Source: Front Nutr. 2023 Jul 18;10:1232424. doi: 10.3389/fnut.2023.1232424 (PMC10400352; doi:10.3389/fnut.2023.1232424)
Supplement: Supplementary file 2 [file Data_Sheet_2.PDF]

Nutritional knowledge questionnaire
# – NUTRIKOB –

Name ……………………………

Surname …………………………

Date of birth ……………………..

Compilation Date ………………..

<u>Section 1: experts' recommendation</u>

1. What is the recommended frequency of consumption of the following foods? (One answer for each food)

	Daily	Weekly	Occasionally	I don't know
Soft drinks	☐	☐	☐	☐
Vegetables	☐	☐	☐	☐
Sauces (es. Mayonnaise)	☐	☐	☐	☐
Red Meat	☐	☐	☐	☐
Whole Foods	☐	☐	☐	☐
Dairy food and processed meat	☐	☐	☐	☐

2. Which milk or yoghurt should be consumed? (Only one answer)

☐ Whole

☐ Low-fat

☐ Both previous answers

☐ Neither dairy products should be avoided

☐ I don't know

3. How many times a week is the consumption of fish recommended? (Only one answer)
   - ☐ 1-2 times a week
   - ☐ 3-4 times a week
   - ☐ Everyday
   - ☐ I don’t know
4. How many times a week is breakfast recommended? (Only one answer)
   - ☐ 3 times a week
   - ☐ 4 times a week
   - ☐ Everyday
   - ☐ I don’t know
5. How many meals is it recommended to consume every day? (Only one answer)
   - ☐ 1-2
   - ☐ 3-5
   - ☐ 6-7
   - ☐ I don’t know
6. In a balanced diet, which meals should include the consumption of vegetable or animal protein?
   - ☐ Breakfast, lunch and dinner
   - ☐ Only dinner
   - ☐ Breakfast and lunch
   - ☐ I don’t know

Section 2: Food groups and nutrients

1. How do you rate the added sugar content of these foods? (One answer for each food)

	High	Low	I don't know
Zero or light beverages	☐	☐	☐
Fruit jam	☐	☐	☐
Plain yogurt	☐	☐	☐
Ketchup	☐	☐	☐
Melon	☐	☐	☐

2. How do you rate the salt content of these foods? (One answer for each food)

	High	Low	I don't know
Fruit	☐	☐	☐
Cassette bread	☐	☐	☐
Ricotta cheese	☐	☐	☐
Meat	☐	☐	☐
Canned foods (tuna-legumes)	☐	☐	☐

3. How do you rate the fiber content of these foods? (One answer for each food)

	High	Low	I don't know
Kiwi	☐	☐	☐
Walnuts	☐	☐	☐
White meat	☐	☐	☐
Eggs	☐	☐	☐
Lentils	☐	☐	☐
Common pasta	☐	☐	☐

4. Do you think these foods are a good source of protein? (One answer for each food)

	Yes	No	I don't know
Chicken	☐	☐	☐
Cheese	☐	☐	☐
Fruit	☐	☐	☐
Beans	☐	☐	☐
Butter	☐	☐	☐
Fish	☐	☐	☐

5. Vitamins and minerals provide the same amount of calories compared to carbohydrates and proteins: (one answer only)
   - ☐ True
   - ☐ False
   - ☐ I don't know
6. The amount of calcium in a glass of whole milk compared to a glass of skim milk is (one answer only):
   - ☐ About the same
   - ☐ Higher
   - ☐ Lower
   - ☐ I don't know
7. Which of the following nutrients is higher in calories for the same weight? (One answer only)
   - ☐ Sugars
   - ☐ Carbohydrates
   - ☐ Proteins
   - ☐ Fats

8. To a person who needs to lose weight would you recommend the use of mineral and vitamin supplements:
   - ☐ True
   - ☐ False
   - ☐ I don't know

<u>Section 3: healthy food choices</u>

1. Which of the following would be a better choice for reducing fat while eating a meal at a restaurant? (One answer only)
   - ☐ Tortellini cream and ham
   - ☐ Risotto with cheeses
   - ☐ Gnocchi with ragù (meat sauce)
   - ☐ I don't know

2. Which of the following would be a better choice for a well-balanced meal? (One answer only)
   - ☐ Roast turkey, mashed potatoes and vegetables
   - ☐ Stew with peas and roasted potatoes
   - ☐ Fried fish
   - ☐ I don't know

3. Which of the following would be a better choice for a "quick meal"? (One answer only)
   - ☐ Pizza slice and cola drink + fruit
   - ☐ Ham and cheese sandwich + skim yogurt + water
   - ☐ Salad and tuna sandwich + juice + water
   - ☐ I don't know

4. In the list of ingredients, the first in the list is always the one that is contained in the largest amount: (one answer only)

□ True

□ False

□ I don't know

5. Light foods are always a better choice:

□ True

□ False

□ I don't know

6. Which of the following dairy products might be a better choice if you want to reduce the amount of fat? (One answer only)

□ Light mozzarella cheese

□ Spreadable cheese

□ Dairy cottage cheese

□ I don't know

7. Of these two hazelnut cream spreads, which one contains a higher amount of sugar? (One answer only)

Hazelnut cream spreads **A**	Hazelnut cream spreads **B**
sugar, palm oil, hazelnut, low-fat cocoa, skim milk powder, lactose, whey powder, emulsifier: lecithin (soy), vanillin	Hezelnut, brown sugar, unsweetened cocoa

□ Cream A

□ Cream B

□ I don't know

Section 4: Relationship between diet, health and disease risk

1. Which of these conditions is related to low dietary fiber consumption (One answer only)

   ☐ Bowel disorders

   ☐ Anemia

   ☐ Dental caries

   ☐ Don't know

2. Which of these diseases is related to high salt consumption? (One answer only)

   ☐ Hypothyroidism

   ☐ Type 1 diabetes

   ☐ Hypertension

   ☐ Don't know

3. Which of these recommendations helps prevent cardiovascular disease? (One answer only)

   ☐ Take multivitamin supplements

   ☐ Eat less blue fish

   ☐ Eat less saturated fat

   ☐ Don't know

4. Eating gluten-free foods helps in weight control:

   ☐ True

   ☐ False

   ☐ Don’t know

5. Which of these recommendations helps prevent dietary diabetes (type 2 diabetes)? (One answer only)

   ☐ Eat less refined foods (white flour, white sugar)

   ☐ Drink more fruit juice

   ☐ Eating more cured meats

- □ Don't know

6. Eating lactose-free cheese helps control blood cholesterol levels:
   - □ True
   - □ False
   - □ Don't know
7. To a person who needs to lose weight would you recommend a gluten-free diet:
   - □ True
   - □ False
   - □ Don't know.
